# Individualized aerobic session modulates key metabolic genes in liver and muscle of male offspring from obese dams

**DOI:** 10.20945/2359-4292-2026-0030

**Published:** 2026-04-01

**Authors:** Paloma Brasilio Villalta, Laís Angélica de Paula Simino, Thais de Fante, Thomaz Ramalheira Guadagnini, Natalia de Almeida Rodrigues, Fúlvia de Barros Manchado Gobatto, Marcio Alberto Torsoni, Adriana Souza Torsoni

**Affiliations:** 1 Laboratório de Distúrbios do Metabolismo (LabDiMe), Faculdade de Ciências Aplicadas, Universidade Estadual de Campinas (Unicamp), Limeira, SP, Brasil; 2 Laboratório de Fisiologia Aplicada ao Esporte, Faculdade de Ciências Aplicadas, Universidade Estadual de Campinas, Limeira, SP, Brasil

**Keywords:** Aerobic exercise, microRNAs, maternal obesity, DOHaD

## Abstract

**Objective:**

Exercise interventions can improve parameters in offspring predisposed to metabolic issues. In this study, we investigate whether acute aerobic exercise in offspring can improve metabolism via miRNA modulation in mice programmed by maternal obesity.

**Materials and methods:**

Female Swiss mice fed either a standard chow (C) or a high-fat diet (HF) during gestation and lactation were mated with C male mice. Offspring fed the C diet underwent swimming exercise protocols, consisting of water adaptation (14 days), a lactate minimum test, and an aerobic intensity exercise session or no exercise at 84 days of age.

**Results:**

Offspring of obese dams (OHF) exhibited increased hepatic glycogen and triglyceride compared to offspring of control dams (OC). However, in offspring of obese dams subjected to an individualized aerobic session (OHF-E), these parameters did not differed from the other groups. Hepatic gene expression analysis showed that *miR-122* was upregulated in OHF-E, inversely to *Agpat* levels. Additionally, OHF exhibited higher *miR-370* and lower *Cpt1a* levels; exercise restored *miR-370* and elevated *Cpt1a* levels in OHF-E. Regarding muscle tissue, exercise reduced *Ptp1b* expression in OHF-E and increased *Hif1a* and *Pparg*, despite no changes observed in *miR-206* levels.

**Conclusion:**

A single session of exercise significantly affected miRNA and transcript levels related to hepatic lipid and muscle glucose metabolism, suggesting that even one bout of exercise can benefit offspring in the context of maternal metabolic programming. This highlights tissue responsiveness and adaptive capacity, warranting further investigation into its potential as a long-term, non-pharmacological intervention.

## INTRODUCTION

The impact of the perinatal environment on offspring’s health is part of the Developmental origins of health and disease (DOHaD) research field, which has been extensively studied for a better understanding of chronic noncommunicable disease genesis (^1^). Maternal obesity during critical developmental periods is related to predisposition to obesity, hypercholesterolemia, hypertension, body weight gain, insulin resistance, and fatty liver in offspring (^2^). Investigations in animal models have demonstrated an association between these alterations and epigenetic modulations in metabolically active tissues during pregnancy and lactation (^3^).

Among all recognized epigenetic mechanisms, changes in the expression of microRNAs (miRNAs) have been extensively studied due to their capacity to regulate gene expression at the post-transcriptional level (^4^). Specific types of miRNAs, including myomiRNAs like *miR-206* and liver-specific miRNAs such as *miR-122* and *miR-370*, are highly abundant in skeletal muscle and liver tissue, respectively, and play essential roles in various biological processes, including muscle regeneration (^5^) and lipid metabolism (^6^).

The literature shows that miRNAs can also be modulated by physical exercise (^6^-^8^). Concomitantly, while not addressed here, the release of trained muscle exosomes carrying a specific miRNA signature could explain, at least in part, the communication between muscle and target tissues during exercise (^9^). Evidence suggests that maternal exercise has a protective effect on metabolic programming and may lead to the reprogramming of the offspring, resulting in long-term benefits such as reduced liver fat accumulation and improved glucose tolerance (^10^). Few studies have shown that early post-weaning training interventions in offspring can improve metabolic parameters impaired by maternal obesity, regardless of nutritional modifications (^11^). However, to our knowledge, there is a gap in the literature regarding whether physical exercise can modify the miRNA expression in offspring promoted by an obesogenic environment during development, potentially representing an early event in the glycemic homeostasis regulation.

Thus, this study aimed to investigate whether a single aerobic session in adult offspring could modulate the expression of miRNAs and their target genes related to lipid metabolism and insulin sensitivity in the liver and muscle, which may be programmed by maternal obesity.

## MATERIALS AND METHODS

### Animals and diet

**Figure 1:**
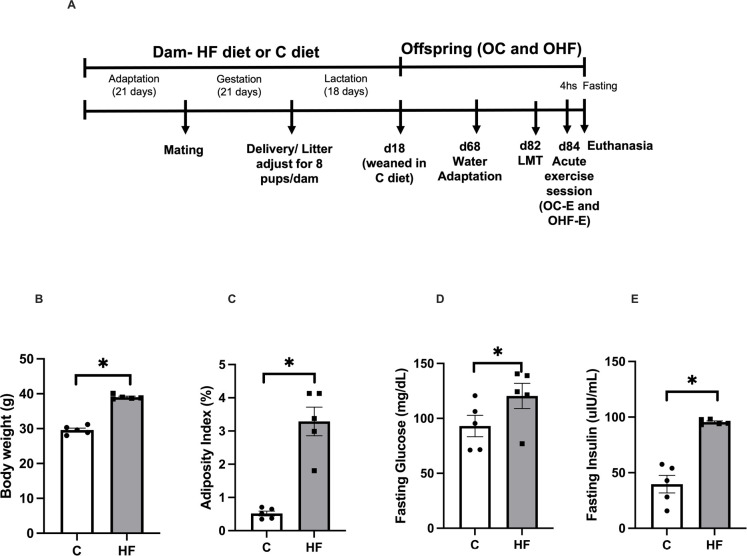
Maternal murinometric and metabolic parameters of HF and C groups. Experimental Design (**A**), maternal body weight (**B**), adiposity index (**C**), fasting glucose (**D**), and insulin (**E**) after weaning. n = 5/group. Student’s t-test. n=5/group. *p<0.05.

In total, 10 five-week-old female Swiss mice were randomly chosen to receive either a standard chow (C, 3.5 kcal/g) or a high-fat diet (HF, F45%, 4.6 kcal/g) (**Supplementary Table 1**) *ad libitum* for an adaptation period of 21 days, and were then mated for 3 days with male mice fed only C (2 females and 1 male per cage). Animals were housed in polypropylene micro-isolators at 22°C ± 1°C with lights on from 06:00 to 18:00 h. The HF diet was prepared as described elsewhere (^3^). Females were fed the same diet as during the adaptation period (C or HF) throughout gestation and lactation (**Figure 1A**).

On the delivery day, litters were adjusted to eight (n = 8) pups per dam. Offspring were weaned at d18 and fed C. On d68, all mice underwent a water adaptation protocol, and by d82, they were subjected to the lactate minimum test (LMT) to determine individual maximal aerobic capacity. Then, two pups from each litter were randomized into exercised and non-exercised groups, giving rise to four groups: non-exercised offspring (OC, n = 5 and OHF, n = 5) and exercised offspring (OC-E, n = 5 and OHF-E, n = 5). A single exercise session was performed at d84. In this case, OC-E and OHF-E were subjected to physical effort at 70% of the LM intensity for 1 hour. After the exercise, the animals were fasted for 4 hours, followed by anesthetic deepening (139.2 mg/bw ketamine, 18.4 mg/bw xylazine, and 4 mg/bw diazepam) and then euthanized by decapitation for sample collection. Maternal data were collected after weaning. Dams underwent 8h of fasting before euthanasia, when the epigonadal white adipose tissue was weighed to measure adiposity, and blood was collected to measure fasting blood glucose and insulin. All procedures complied with the ethical standards of the Brazilian national guidelines on the care and use of laboratory animals. The study was approved by the Research Ethics Committee on the Use of Animals (Protocol Number 3913-1). Metabolic assessments and molecular evaluations were conducted independently and in a blinded manner to reduce bias.

### Adaptation to water

Individual adaptation to the aquatic environment began at d68 and lasted 14 days to minimize stress, as described elsewhere (^12^). This involved the progressive exposure of all mice from each group to water temperatures of 31±1°C in polyvinyl chloride tanks (30 cm diameter × 100 cm depth) with smooth surfaces to prevent mice from resting on the bottom of the tank. Briefly, mice were placed in shallow water (3 cm deep) for 15 min/day over the first three days. By the fourth day, they transitioned to swimming in deep water (100 cm deep) for 2 min, with an increment of 2 min/day until the eighth day of the procedure. From the ninth day, mice swam in deep water with a progressively increasing load (3%-15% bw) tied to the back of each animal, for periods varying from 5 minutes to 30 seconds over the course of six days, always followed by another 5-min session without load. Since the adaptation protocol was performed individually, while one group of animals underwent the adaptation protocol or exercise, the remaining groups were housed in their usual cages with food *ad libitum*.

### Lactate minimum test (LMT) and individualized exercise session in offspring

**Supplementary Figure 1:**
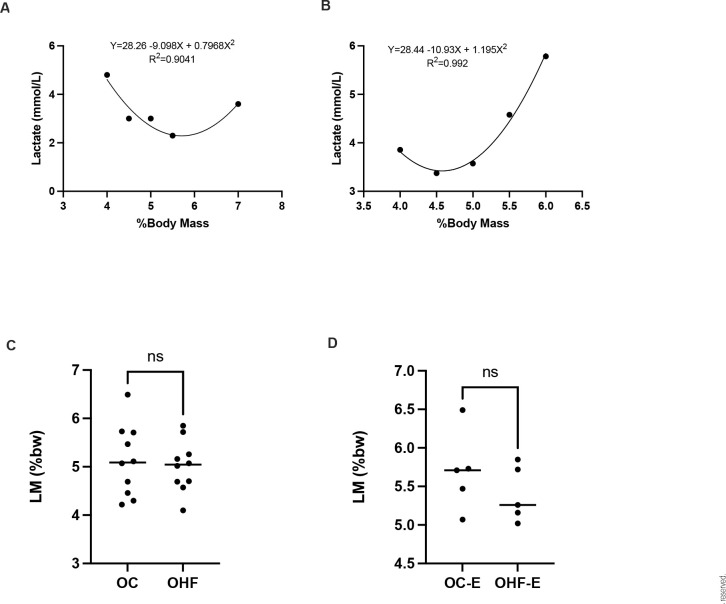
Comparison of the intensity of lactate minimum (LM) during incremental phase. Example curve of blood lactate concentration (mmol·L^-1^) versus intensity (percentage of body mass (% bm)) during the stages (5 min) of the incremental phase of OC mice (**A**), Example curve of blood lactate concentration (mmol·L^-1^) versus intensity (percentage of body mass (% bm)) during the stages (5 min) of the incremental phase of OHF mice (**B**). Intensity of LM of all mice from OC and OHF groups (**C**), Intensity of LM of mice from groups randomized to exercise -OC-E and OHF-E (**D**). Intensity of LM (%bw) expressed as mean. ns, not statistically significant.

The maximal aerobic capacity for each animal in all groups was determined by performing the LMT, as previously described (^12^). Briefly, LMT was conducted in three steps: i) induction of hyperlactatemia, performed by a 30-second swimming effort with a 13% body weight (bw) load, followed by 30 seconds of rest, and then a second effort with 13% bw load until exhaustion; ii) a 9-min passive recovery period to allow the accumulation of lactate in the bloodstream; and iii) an incremental phase, in which 4.0, 4.5, 5.0, 5.5, 6.0, and 7.0% bw loads were carried for 5 min, with a 30-second interval between efforts, and blood samples collected to determine lactatemia during the incremental protocol. The maximal aerobic capacity (i.e., anaerobic threshold intensity [AnT]) determined by LMT was individually obtained as the intensity relative to a point derived from a second order polynomial curve (R^2^ > 0.8). An example of the curve from OC and OHF mice is shown in **Supplementary Figure 1A-B**, respectively.

The acute exercise session was conducted in the morning of d84. It consisted of one hour of swimming, with a load corresponding to 70% of the lactate minimum intensity previously determined by the LMT. Due to the high bias of the LMT in estimating individual aerobic capacity for female mice (^12^), only male offspring were evaluated.

### Murinometric and biochemical analysis

Body weight and food consumption were measured weekly. Following euthanasia, the epididymal and retroperitoneal white adipose tissue depots were carefully dissected and weighed separately. The adiposity index was estimated as the ratio of the combined weight of these fat pads to the final body weight. Fasting glucose was determined using an Accu-Chek Performa glucometer (Roche Diagnostics). Hepatic total lipids were measured by the Folch method, as previously described in Panzarin and cols. (^13^), and hepatic glycogen was evaluated as described in Fante and cols. (^14^).

### *In silico* analysis of miRNAs predicted targets

*In silico* analysis was performed to identify predicted target genes in *Mus musculus* corresponding to miRNAs previously identified as altered in the offspring of obese dams, specifically *miR-370, miR-122*, and *miR-206* (^15^). The prediction of miRNA/mRNA targets was conducted using the TargetScan platform, version 8.0 (https://www.targetscan.org/vert_80/).

### mRNA and microRNA analysis

Total RNA and miRNA from the liver and soleus muscle were extracted from about 150 mg of tissue using RNAzol RT (MRC, US), following the manufacturer’s recommendations. cDNA was obtained as described elsewhere (^3^). Relative miRNA or gene expression was determined using the Taqman detection system and primers for *miR-122-5p* (ID 002245), *miR-370-3p* (ID 002275), *miR-206* (ID 000510), *Agpat1* (Mm00479699_g1), *Acadvl* (Mm00444293_m1), *Cpt1a* (Mm01231183_m1), *Hif1a* (Mm00468869_m1), *Pparg* (Mm00440940_m1), and *Ptp1b* (Mm00448427_m1). *U6snRNA* (ID 001973) or β*actin* (Mm02619580_g1) were used as endogenous controls (Thermo Fisher Scientific, US). qRT-PCR was performed on the ABI Prism 7500 Fast, and data were expressed as relative values determined by the comparative threshold cycle (Ct) method (2-∆∆Ct).

### Statistical analysis

Results are expressed as mean ± standard error. The GraphPad Prism (Version 10.0.0, GraphPad Software Inc., San Diego, CA, US) was used for data analysis. To determine outliers, the Grubbs’ method was applied. The normality of the data was tested using the Shapiro-Wilk test. Student’s *t*-test was used to compare two groups, while two-way analysis of variance (ANOVA) was used for multiple comparisons A post hoc test (Šidák) was applied only when an interaction with a p-value < 0.05 was detected to determine a significance level of p < 0.05. The significance indicators of each graph were specified in the respective figure legend.

## RESULTS

### The aerobic session improved some metabolic parameters from offspring of obese dams

The maternal obesity phenotype of HF dams was confirmed by increased body weight, adiposity, fasting glucose, and insulin measurements compared to C (**Figure 1B-E**).

**Figure 2:**
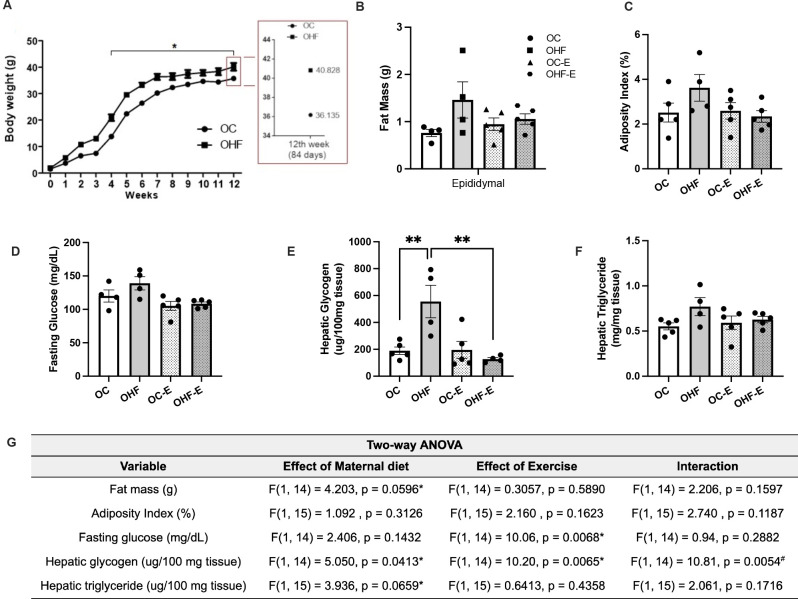
The acute aerobic exercise session ameliorates metabolic parameters in offspring. Body weight (**A**), fat mass (**B**), adiposity index (**C**), fasting glucose (**D**), hepatic glycogen (**E**), and triglyceride (**F**) of adult non-exercised offspring from control (OC) and obese (OHF) dams, and adult exercised offspring from control (OC-E) and obese (OHF-E) dams. ANOVA table results (**G**). post hoc test (Sidák). n=5/group. *p<0.05; **p<0.01.

Offspring of obese dams (OHF) had higher body weight (1.13-fold) and increased hepatic glycogen (2.6-fold) compared to offspring of control dams (OC). Maternal high fat diet (HF) also seemed to impact fat mass (p = 0.0596) and hepatic triglyceride content (p = 0.0659) at d84, although no differences were observed in food intake (25.1 ± 2.075, OHF vs 24 ± 1.25 kcal/day, OC) (**Figure 2A-F**). Additionally, aerobic capacity did not differed between mice from the OC and OHF groups, including those that were randomized for exercise (OC-E and OHF-E) (**Supplementary Figure 1C-D**).

Conversely, fasting glucose was affected by exercise although without interference from the maternal diet (**Figure 2D**). OHF-E presented lower hepatic glycogen than OHF (4.37-fold), while fat mass, adiposity index, and triglycerides presented no significant changes (**Figures 2B-C, E-F**).

### Aerobic exercise modulated key hepatic and muscle microRNAs in the offspring of obese dams

Previous studies have demonstrated that offspring exposed to maternal obesity during gestation and/or lactation experience alterations in hepatic microRNAs that disrupt energy and lipid homeostasis, notably with the upregulation of *miR-370* and downregulation of *miR-122* (^3^,^16^). Moreover, studies reported that *miR-206* levels can significantly change after a single exercise session, with effects lasting up to 24 hours (^17^).

**Figure 3:**
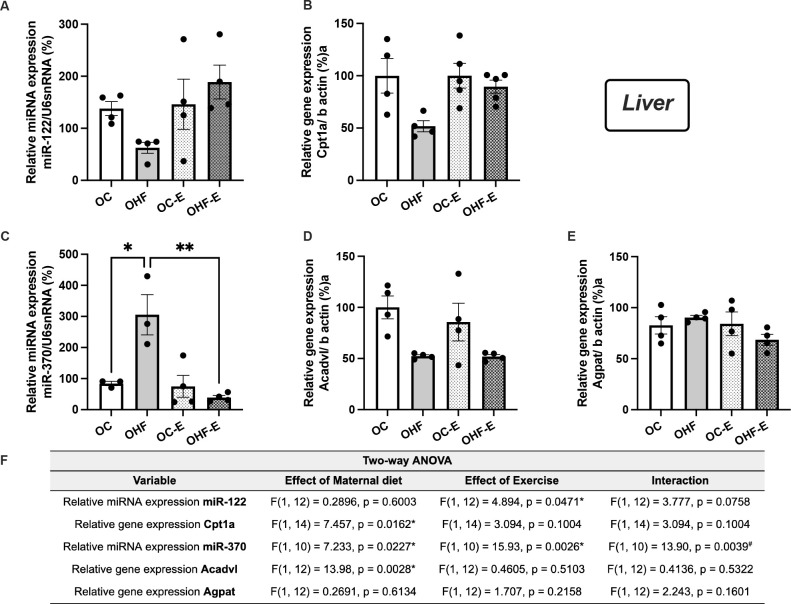
Key hepatic miRNAs and genes related to lipid metabolism modulated by the acute aerobic exercise sessions in offspring. qRT-PCR of hepatic *miR-122* (**A**), *Cpt1a* (**B**), *miR-370* (**C**), *Acadvl* (**D**), and *Agpat* (**E**) of adult non-exercised offspring from control (OC) and obese (OHF) dams, and adult exercised offspring from control (OC-E) and obese (OHF-E) dams. ANOVA table results (**F**). post hoc test (Sidák). n=3-5/group. *p<0.05; **p<0.01.

**Figure 4:**
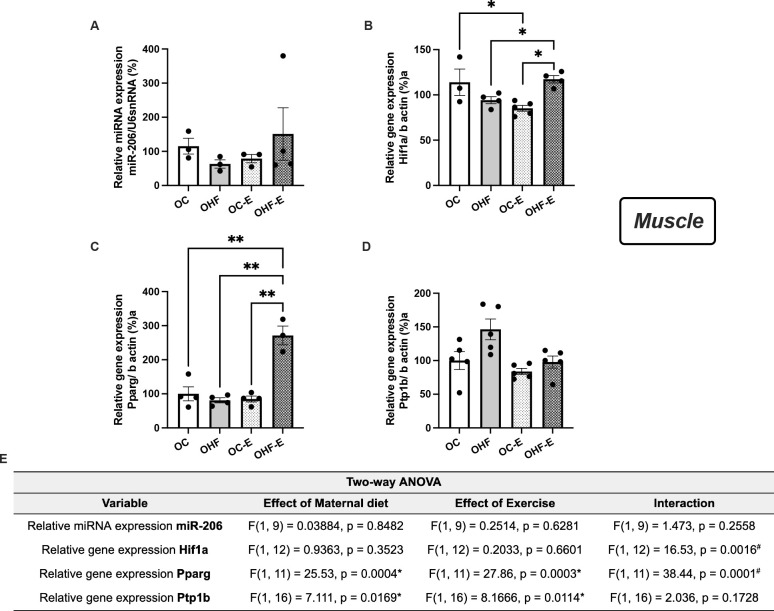
Key muscular miRNA and genes related to insulin sensitivity and mitochondrial biogenesis modulated by the acute aerobic exercise sessions in offspring. qRT-PCR of muscular *miR-206* (**A**), *Hif1a* (**B**), *Pparg* (**C**), and *Ptp1b* (**D**) of adult non-exercised offspring from control (OC) and obese (OHF) dams, and adult exercised offspring from control (OC-E) and obese (OHF-E) dams. ANOVA table results (**E**). ost hoc test (Sidák). n=3-5/group. *p<0.05; **p<0.01.

Our analysis revealed that exercise have a significant impact on *miR-122* expression (p = 0.0471) (Figure 3A) in the liver. Nonetheless, OHF showed elevated *miR-370* levels (3.0-fold) compared to OC, and these levels were normalized following an exercise session in OHF-E (p = 0.0011) (**Figure 3C**). In muscle tissue, *miR-206* was not different between groups (**Figure 4A**).

*In silico* analysis identified predicted targets for *miR-122, miR-370*, and *miR-206* in *Mus musculus*. While hepatic miRNAs (*miR-122* and *miR-370*) may target genes important for lipid metabolism, muscle *miR-206* may influence genes related to insulin sensitivity and mitochondrial biogenesis (**Table 1**).

**Table 1:** Bioinformatic analysis by searching for mouse *miR-122-5p, miR-370-3p*, and *miR-206*, and their putative mRNA targets.

microRNA	Target mRNA	Pairing sequence^*^		References (PMID)^**^
**miR-122-5p**	**Agpat**	Position 134-140 of AGPAT1 3’ UTR mmu-miR-122-5p Position 190-196 of AGPAT1 3’ UTR mmu-miR-122-5p Position 461-467 of AGPAT1 3’ UTR mmu-miR-122-5p	5’...UUCUGAAGUGAAUGUCACUCCAU... 3’GUUUGUGGUAACAGUGUGAGGU 5’ ...CGUGGGUGCAGUCUCCACUCCAA... 3’GUUUGUGGUAACAGUGUGAGGU 5’ ...UGGAAGCUGCACCUGACACUCCU... 3’ GUUUGUGGUAACAGUGUGAGGU	22820288 35048271 28239403
**miR-370-3p**	**Cpt1a**	Position 552-558 of CPT1A 3’ UTR mmu-miR-370-3p	5’ ...UCCACAUUUCCUGGA-AGCAGGAU... 3’ UGGUCCAAGGUGGGGUCGUCCG	20124555 24666709 35048271 28239403
**miR-206**	**Hif1a**	Position 3466-3472 of HIF1A 3’ UTR hsa-miR-206	5’ ...UAACCUCACGAUUAUCAUUCCAA... 3’ GGUGUGUGAAGGAAUGUAAGGU	23628900 30250188 31880296
	**Ptp1b**	Position 1172-1178 of PTPN1 3’ UTR hsa-miR-206	5’ ...GGGAUCAGCCUCCGCCAUUCCAA... 3’GGUGUGUGAAGGAAUGUAAGGU	31048362 31695578 31894853

* According to TargetScan release 8.0 (https://www.targetscan.org/vert_80/).

** PubMed Identifier.

Specifically, *miR-122-5p* is predicted to target 1-acylglycerol-3-phosphate O-acyltransferase 1 (*Agpat*), which encodes an enzyme crucial for triglyceride synthesis in the liver, at three different sites on the mature mRNA sequence. Concurrently, *miR-370-3p* is predicted to target the *Cpt1a* mRNA, encoding carnitine palmitoyl transferase, a protein essential for mitochondrial fatty acid transport and oxidation (**Table 1**).

Bioinformatic analysis also revealed two mRNA pairing sequences for miR-206: one corresponds to the gene encoding the alpha subunit of the transcription factor hypoxia-inducible factor-1 (HIF-1), a critical regulator of cellular responses to hypoxia, and the other targets the protein-tyrosine phosphatase PTP1B, which negatively regulates insulin signaling (**Table 1**).

The modulation of hepatic and muscle microRNAs in the offspring of obese dams by acute aerobic exercise resulted in changes to their predicted mRNA targets.

The bioinformatic analysis was validated using qPCR with specific primers for predicted target genes, as well as those identified in the literature as potential targets.

Liver data indicated an effect of maternal HF diet on *Cpt1a* levels (p = 0.0162) and *Acadvl* (p = 0.0028), paralleling the increase in *miR-370* (**Fig. 3B-D**). Although *Acadvl* (Acyl-CoA Dehydrogenase Very Long Chain) is not a predicted target of *miR-370-3p*, previous findings show an inverse relationship between hepatic *miR-370* and *Acadvl* expression in offspring of obese dams (^3^,^13^,^16^), which justified its inclusion in the evaluation.

In muscle tissue, the exercise session induced an increase in *Hif1a* expression (28%) in the OHF-E group compared to OHF and OC-E (**Figure 4B**). Moreover, the exercise promoted an increase in *Pparg* levels (2.7-fold) compared to both non-exercised OC and OHF and OC-E (**Figure 4C**). Additionally, *Ptp1b*, a predicted target of *miR-206,* presented an effect of both maternal HF diet (p = 0.0169) and exercise (p = 0.0114), although no interaction was observed between them (**Figure 4D**).

## DISCUSSION

The influence of physiological and environmental conditions during pregnancy and early postnatal development on lifelong health trajectories and disease susceptibility has been well-established in scientific literature (^18^). Recent findings emphasize that even brief exercise interventions can produce lasting metabolic benefits (^19^). This is particularly relevant in maternal obesity, which is linked to adverse metabolic programming outcomes in offspring.

**Supplementary Figure 2:**
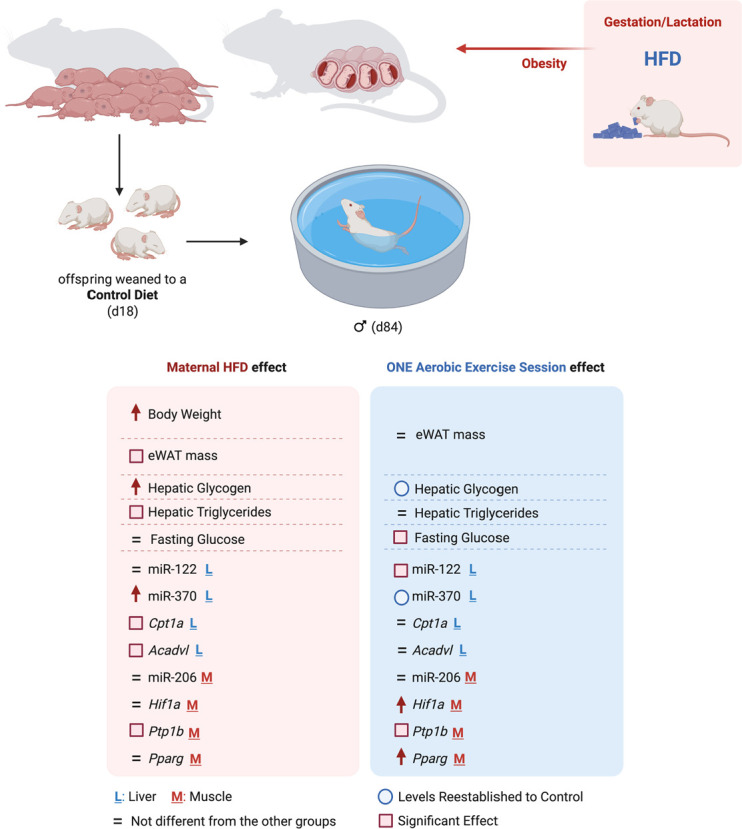
Graphical abstract.

Our investigation focused on whether an aerobic exercise session in adulthood could modulate the expression of key hepatic and muscle genes involved in lipid metabolism and insulin sensitivity in offspring of obese dams. Remarkably, we found that even a single session of exercise can modulate microRNA expression, impacting target genes within these tissues and leading to improvements in metabolic parameters in offspring (**Supplementary Figure 2**).

Consistent with prior research, our results confirmed that maternal obesity correlates with increased body weight in offspring from early life (^20^). The pronounced weight gain observed in offspring of obese dams (OHF) from the fourth week underscores the propensity for early excessive weight gain driven by maternal obesity.

While existing studies demonstrated that interventions during pregnancy or lactation, such as nutritional or exercise modifications, can improve offspring outcomes (^21^), there is limited investigation into postnatal interventions after the establishment of a programmed phenotype. Our findings suggest that even a single session of swimming may transiently counteract some metabolic disturbances associated with maternal obesity, highlighting its potential as a modifiable stimulus.

In contrast, a study by Kasch and cols. (^22^) reported that voluntary wheel running was ineffective in reducing weight gain or reversing impaired glucose metabolism gene expression in the skeletal muscle of offspring from high-fat diet (HF)-fed dams, indicating a potential limitation in training efficacy. However, in our study, a session of swimming exercise provided significant benefits to offspring of obese dams (OHF-E), including improved biochemical parameters such as hepatic glycogen, bringing them in line with control groups (OC and OC-E). Similar improvements after acute resistance or aerobic exercise have been reported by others (^23^,^24^). This highlights that targeted exercise can reduce, at least temporarily, metabolic dysfunctions caused by maternal obesity.

The association of maternal obesity with an increased risk of metabolic dysfunction-associated steatotic liver disease (MASLD) and its progression to metabolic dysfunction-associated steatohepatitis (MASH) in offspring is especially concerning (^25^,^26^). In the liver, alterations in the expression of *miR-122* - the most abundant miRNA - and *miR-370*, highlighted in our findings, are associated with changes in hepatic lipid metabolism and are known to be implicated in MASLD and MASH (^3^). Through *in silico* predictions and qPCR validations, we identified mRNA targets influenced by exercise-modulated miRNAs that are closely associated with pathways driving triglyceride synthesis and hepatic fatty acid metabolism (^27^,^28^). Previously, our group demonstrated that offspring of obese dams displayed increased hepatic lipid accumulation due to the upregulation of *miR-370* and lipogenic genes like *Agpat*, alongside the downregulation of *miR-122* and oxidative genes, such as *Acadvl* and *Cpt1a* (^3^). Here, we demonstrate that exercise induces upregulation of hepatic miR-122. Additionally, the elevated hepatic miR-370 observed in OHF was normalized following exercise in OHF-E. Although Cpt1a, which was downregulated by maternal diet, was not influenced by exercise and direct measurements of lipid oxidation were not conducted, it is plausible that the exercise-induced changes in hepatic microRNA expression may contribute to the restoration of lipid oxidation capacity.

Likewise, prolonged swimming exercise, combined or not with intermittent fasting, has been shown to improve blood glucose and insulin levels and effectively combat MASLD in diet-induced obesity models, modulating *miR-122-5p* and subsequently reducing the expression of lipid synthesis-related genes (*Srebp-1c, Fasn*, and *Acc1*) while enhancing genes related to fatty acid oxidation (*Cpt1a*) (^29^). Although our intervention was conducted in a distinct animal model and did not constitute a long-term training protocol, the acute exercise response illustrates potential similarities with chronic responses. Adjustments in both intervention duration and frequency could reveal more significant benefits in offspring hepatic lipid metabolism.

Exercise is also known to influence microRNA profiles in skeletal muscle, leading to improved glucose metabolism and insulin sensitivity (^8^). In skeletal muscle, *miR-206* has emerged as a biomarker and potential therapeutic target (^7^). *miR-206*, a highly conserved muscle-specific microRNA essential for myogenic differentiation (^28^), is upregulated after acute aerobic exercise (^15^,^17^) and a single session of resistance training, with changes detectable for up to 24 hours, including significant alterations within the first hour post-exercise (^15^). Conversely, high-fat diet intake suppresses *miR-206*, a change linked to impaired vascular reactivity and muscle function (^7^). Moreover, its overexpression has been shown to inhibit lipogenesis and triglyceride secretion (^8^). In our study, although *miR-206* levels did not differ in offspring of obese dams, bioinformatic predictions indicated that it influences genes related to insulin sensitivity and mitochondrial biogenesis.

*miR-206* is known to target *Hif1a* (^30^), a key regulator of glycolysis, as well as *Ptp1b* (^31^), a negative modulator of insulin signaling. Furthermore, it appears to have a positive correlation with *Pparg* (^32^), which plays a significant role in increasing insulin sensitivity and promoting endogenous adiponectin production, thereby exerting a protective effect against diet-induced insulin resistance. Our findings indicate that a single exercise session significantly increased *Hif1a* expression in offspring of obese dams subjected to exercise (OHF-E) compared to non-exercised OHF. Moreover, we observed elevated *Pparg* levels in OHF-E relative to both non-exercised offspring (OC and OHF) and exercised control (OC-E). Notably, *Ptp1b* was impacted by both maternal HF diet and exercise. The concurrent upregulation of *Hif1a* and *Pparg* post-exercise, alongside the effects on *Ptp1b* levels, highlights the diverse metabolic health benefits conferred by acute physical activity.

The absence of differences in the reciprocal modulation of miRNA and some predicted targets may be due to the low sample size, which is a limitation of the study. Although miR-206 levels remained unchanged, exercise modulated its target genes, *Ptp1b, Pparg,* and *Hif1a,* illustrating the dynamic, time-sensitive nature of microRNA regulation. Conversely, the relationship between *miR-122* and *Agpat* exhibited the opposite pattern. Target mRNAs can respond quickly via translational repression or destabilization, whereas mature miRNAs are relatively stable, creating a temporal mismatch between gene and miRNA expression changes (^33^).

Fante and cols. (^14^) reported that offspring of obese dams displayed pronounced insulin resistance in the soleus skeletal muscle, evidenced by elevated levels of PEPCK and PTP1B along with reduced p-IRS1 and p-AKT in response to insulin stimulation. Although our study did not assess the long-term effects of exercise, the acute modulation observed was sufficient to reduce *Ptp1b* gene expression and decrease fasting glucose. Given the critical roles of myokines and other signaling pathways activated during physical activity, a comprehensive analysis of these factors, in conjunction with epigenetic modifications, will be essential for developing effective strategies to combat obesity and related metabolic disorders, with implications that may extend across generations.

In summary, our findings suggest that a single session of swimming may transiently counteract some metabolic alterations associated with maternal obesity, highlighting its potential as an initial modulating stimulus. However, it is unlikely that these changes observed after a single exercise session persist for an extended period unless reinforced by repeated exercise stimuli. Nevertheless, they may represent a molecular signature that signals the tissue’s responsiveness to exercise and its capacity for metabolic adaptation. The long-term benefits of such interventions require further investigation, including assessments of the gain and loss of function of miRNA modulated by exercise. Since outcomes may differ between sexes, future studies should include female subjects. Although one limitation is that a group of mice was kept fed in their cages while others underwent training, we believe this had minimal impact on the results, as exercise sessions occurred in the morning, a period typically characterized by reduced food intake.

Further studies should focus on elucidating the sustainability of these molecular changes and uncovering the underlying mechanisms of exercise-induced reprogramming, with the goal of establishing robust, lifelong strategies against metabolic disorders arising from maternal dietary influences.

## Figures and Tables

A single individualized aerobic exercise session can ameliorate metabolic parameters, microRNAs and target-genes expression in liver and soleus muscle of offspring programmed by maternal obesity.

## Data Availability

datasets related to this article will be avail-able upon request to the corresponding author.

## SUPPLEMENTARY MATERIALS

**Supplementary Table 1:** Nutritional composition of the experimental diets

	Chow Diet ^*^	HFD# (45% kcal from fat)
Net Protein (g %)	20.0	23.3#
Fat content (g %)	4.0^*^	24.0^*^#
Carbohydrates (g %)	65.95^*^	42.1#
Fiber (g %)	5.0	5.55
Mineral Mix (g %)	3.5	3.5
Vitamin Mix (g %)	1.0	1.0
Choline	0.25	0.25
Cystine	0.3	0.3
Total	100.0	100.0
Energy (kj/g)	14.6	19.3

* NUVILAB^®^ Cr-1; Nuvital .

#Based on AIN-93G (^34^) - AIN-93G was modified to contain a higher fat content (45%) than control diet and a higher net protein than AIN-93M. It is indicated during pregnancy, lactation and growth period in rodents.

* Soy oil and #lard.

* #Starch and sucrose.
